# Numerical Study on the Effect of Pre-Strain on Detwinning in Rolled Mg Alloy AZ31

**DOI:** 10.3390/ma14206069

**Published:** 2021-10-14

**Authors:** Chao Ma, Xing Duan, Xiaoqian Guo, Hua Qiao, Lianying Zhang, Peidong Wu

**Affiliations:** 1 School of Physics and New Energy, Xuzhou University of Technology, Xuzhou 221000, China; mbqq2008@126.com (C.M.); zhanglianying@126.com (L.Z.); 2 School of Mechanics and Civil Engineering, China University of Mining and Technology, Xuzhou 221116, China; TS20030002A31@cumt.edu.cn; 3 Department of Mechanical Engineering, McMaster University, Hamilton, ON L8S 4L7, Canada; peidong@mcmaster.ca

**Keywords:** pre-straining, detwinning, crystal plasticity, magnesium alloy

## Abstract

The deformation behavior of rolled Mg alloy AZ31, previously compressed along the rolling direction (RD), was numerically investigated under reverse tension. The EVPSC-TDT model was employed to study the effect of pre-strain on detwinning for 3%, 6% and 9% pre-compressed materials along the RD. A new criterion was proposed to control the exhaustion of detwinning under reverse tension. Numerical results show good agreement with the corresponding experimental data. It was demonstrated that the proposed criteria can capture the key features associated with detwinning in pre-compressed materials. Regardless of the amount of pre-compression, detwinning is activated under reverse tension, leading to low yield stress and a typical s-shaped flow curve. The inflection point reflects the exhaustion of detwinning, which is delayed when increasing the amount of pre-compression.

## 1. Introduction

It is well known that the applications of magnesium alloys are restricted because of their high directional anisotropy and poor room temperature formability [1,2]. Due to the low crystallographic symmetry and limited slip systems activated at room temperature, the $\left\{10\bar{1}2\right\}$ extension twinning plays an important role in accommodating plastic deformation. The $\left\{10\bar{1}2\right\}$ extension twinning dominates the initial plastic deformation of Mg alloys under tension parallel to the *c*-axis of the HCP lattice or compression perpendicular to the *c*-axis [3,4]. The extension twinning results in a 86.3° reorientation of the basal pole. Due to this nearly 90° reorientation, detwinning could occur in the twinned areas at subsequent reversed loading [5,6,7,8,9,10]. Very intensive experimental studies have been conducted to investigate the role of twinning and detwinning under cyclic loading and strain-path change [5,6,7,8,9,10]. Detwinning as a reverse process of twinning is different from twinning in some respects. There is no nucleation in the process of detwinning, and the activated stress required for detwinning is lower than that for twin nucleation but larger than that for twin growth [11].

Previous experimental reports indicate that pre-twinning, which produces $\left\{10\bar{1}2\right\}$ twin lamellar structure, has an obvious effect on yield stress, peak stress, elongation and tension–compression asymmetry of Mg alloys, including extruded AM30 [2,12,13], rolled AZ31 [14,15,16,17] and extruded AZ31 [5,18,19,20]. In addition, the anisotropic behavior of Mg alloys is also influenced by the $\left\{10\bar{1}2\right\}$ twin structure produced during pre-strain deformation. It has been demonstrated that the deformation characteristics of pre-deformed Mg alloys, such as detwinning [1,18,19] and $\left\{10\bar{1}2\}-\{10\bar{1}2\right\}$ double twin [1,14,15], are significantly dependent on the previous loading direction and the amount of the pre-strain.

Based on the predominant twin reorientation (PTR) scheme [21], several crystal plasticity-based approaches for modeling the twinning and detwinning behavior of textured Mg alloys have been proposed [22,23,24,25,26,27,28]. On the other hand, Wang et al. [27,28] developed a physical-based constitutive model to describe twinning and detwinning (TDT) deformation behavior, which has been implemented in the Elastic Visco-Plastic Self-Consistent (EVPSC) model [29]. In the TDT model, any new generated twin variant is treated as a new grain, while the PTR model reorients the parent grain into the orientation of its predominant twin variant once the twin volume fraction (VF) of the grain reaches a threshold value. One can refer to [30] for more details on the comparison of the PTR and TDT schemes. The EVPSC-TDT model has been used widely to numerically study the twinning and detwinning behavior of Mg alloys under loadings with strain-path change [10,30,31,32,33] and cyclic loadings (including cyclic shear [34], cyclic tension–compression/compression–tension [27,28,35,36]).

In this study, the deformation behavior of a rolled Mg alloy under reverse tension following pre-compression was investigated by the EVPSC-TDT model. Three specimens with different amounts of pre-compression along the RD were used to study the effect of pre-strain on detwinning during subsequent reverse tension. The paper is organized as follows. Section 2 briefly introduces the EVPSC-TDT model. Numerical results with an emphasis on the effect of pre-strain on detwinning are compared with corresponding experimental data in Section 3. Conclusions are drawn in Section 4.

## 2. Constitutive Model

The EVPSC model has been successfully used to simulate the large strain behavior of HCP [30,31,32,33,34,35,36] and FCC [37,38] polycrystalline materials under different deformation processes. Here, we briefly recapitulate the TDT model. For details, one can refer to Wang et al. [27,28].

The TDT model assumes that a grain has four potential operations associated with twinning and detwinning. Operation A is twin nucleation and initiates a twin band or ‘child’. Operation B is a propagation of the child into the parent grain. Operations A and B increase the twin volume fraction and thus correspond to twinning. Operation C is a propagation of the parent into the child. Operation D is shrinking of the twinned region through twin nucleation within the twin (re-twinning). Operations C and D decrease the twin volume fraction and thus correspond to detwinning. It is clear that Operations B, C, and D are impossible in a twin-free grain [27].

The net evolution of twin volume fraction associated with twinning system β is governed by

(1) $${\dot{f}}^{\beta }=f^{0}({\dot{f}}^{\beta A}+{\dot{f}}^{\beta C})+f^{\beta }({\dot{f}}^{\beta B}+{\dot{f}}^{\beta D})$$

where $f^{0}$ is the volume fraction of the parent, i.e., $f^{0}=1-f^{tw}=1-\sum_{\beta }f^{\beta }$, and superscripts A, B, C and D represent Operations A, B, C and D, respectively.

In the original TDT model, it was assumed that detwinning could be activated only after a twin β has been formed and detwinning ceases once the volume fraction of the twin, $f^{\beta }$, reaches the prescribed residual twin VF, $f_{res}^{\beta }$. A constant value for $f_{res}^{\beta }$ was used in the original TDT model; however, based on the work of Yu et al. [25,26], a new equation describing the residual twin volume fraction during reverse tension is proposed in the present work:

(2) $$f_{res}^{\beta }=a_{twin}^{\beta }\cdot f_{\max}^{pre}\cdot \left[1-\exp(-\frac{f_{\max}^{pre}}{b_{twin}^{\beta }})\right]$$

where $f_{\max}^{pre}$ is the maximum twin VF of a grain during pre-compression. $a_{twin}^{\beta }\cdot f_{\max}^{pre}$ can be regarded as the saturated residual twin VF, and it cannot be larger than $a_{twin}^{\beta }$ as the maximum value of $f_{\max}^{pre}$ is 1.0, indicating that $a_{twin}^{\beta }$ is the maximum value of saturated residual twin VF. $b_{twin}^{\beta }(=b_{twin})$ is a material parameter governing the rate of saturation for each twinning system β. It can be seen that $f_{res}^{\beta }$ is proportional to $a_{twin}^{\beta }$ and $f_{\max}^{pre}$ but inversely proportional to $b_{twin}^{\beta }$. For simplicity, the values of $a_{twin}^{\beta }$ and $b_{twin}^{\beta }$ for all twinning systems are assumed to be the same, denoted as $a_{twin}$ and $b_{twin}$, respectively.

In addition, a threshold twin volume fraction is introduced in the model to terminate twinning because it is rare that a grain can be fully twinned. Similar to the PTR model, the TDT model also introduces two statistical variables: accumulated twin fraction $V^{acc}$ and effective twinned fraction $V^{eff}$. More specifically, $V^{acc}$ and $V^{eff}$ are the weighted volume fraction of the twinned region and volume fraction of twin terminated grains, respectively. The threshold volume fraction $V^{th}$ is defined as

(3) $$V^{th}=\min\{1,B_{1}+B_{2}\frac{V^{eff}}{V^{acc}}\}$$

where $B_{1}$ and $B_{2}$ are two material constants. $B_{1}$ essentially controls the level of strain which a grain can undergo prior to the twinning mechanism beginning to undergo exhaustion; $B_{2}$ controls the rate at which this exhaustion takes place [35].

Finally, for both slip and twinning, the evolution of the critical resolved shear stress (CRSS) $\tau_{cr}^{\beta }$ is provided by

(4) $${\dot{\tau }}_{cr}^{\beta }=\frac{d{\hat{\tau }}^{\beta }}{d\Gamma }\sum_{\chi }h^{\beta \chi }\left|{\dot{\gamma }}^{\chi }\right|$$

where $\Gamma =\sum_{\beta }\int \left|{\dot{\gamma }}^{\beta }\right|dt$ is the accumulated shear strain in the grain, and $h^{\beta \chi }$ are the latent hardening coupling coefficients, which empirically account for the obstacles on system β associated with system χ. ${\hat{\tau }}^{\beta }$ is the threshold stress and is characterized by

(5) $${\hat{\tau }}^{\beta }=\tau_{0}^{\beta }+(\tau_{1}^{\beta }+h_{1}^{\beta }\Gamma )(1-\exp(-\frac{h_{0}^{\beta }}{\tau_{1}^{\beta }}\Gamma ))$$

where $\tau_{0}$, $h_{0}$, $h_{1}$ and $\tau_{0}+\tau_{1}$ are the initial CRSS, initial hardening rate, asymptotic hardening rate, and back-extrapolated CRSS, respectively.

## 3. Results and Discussion

**Figure 1:**
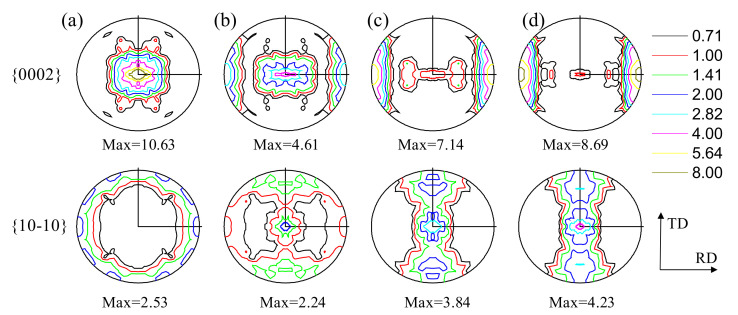
The initial texture and the predicted deformed textures under uniaxial compression along the RD, in terms of {0002} and {10-10} pole figures: (**a**) as-rolled (0% pre-compressed), (**b**) 3% pre-compressed, (**c**) 6% pre-compressed, (**d**) 9% pre-compressed materials.

The material investigated in this study was a hot rolled AZ31 Mg alloy plate which was experimentally studied by Park et al. [1]. The initial texture of the as-received AZ31 plate is represented by 1000 discrete orientations, as shown in Figure 1a. It is a typical strong basal texture with a randomly oriented a-axis in the rolling plane.

In all the simulations reported in the present paper, the reference slip/twinning rate ${\dot{\gamma }}^{0}$ and the rate sensitivity m were prescribed to be the same for all slip/twinning systems: ${\dot{\gamma }}^{0}=0.001\;s^{-1}$ and m=0.05, respectively [30,31,32,33,34,35]. The room temperature elastic constants of the magnesium alloy were assumed to be $C_{11}=58.0$, $C_{12}=25.0$, $C_{13}=20.8$, $C_{33}=61.2$ and $C_{44}=16.6\;\mathrm{GPa}$ [39]. The applied strain rate was $5\times 10^{-3}\;s^{-1}$. Here, the plastic deformation was assumed to be accommodated by basal ($\{0001\}<11\bar{2}0>$), prismatic ($\{10\bar{1}0\}<11\bar{2}0>$) and pyramidal <c+a> ($\{11\bar{2}2\}<11\bar{2}3>$) slips, as well as tensile twinning ($\{10\bar{1}2\}<10\bar{1}1>$). In addition, the affine self-consistent scheme was applied for all the simulations [40].

**Figure 2:**
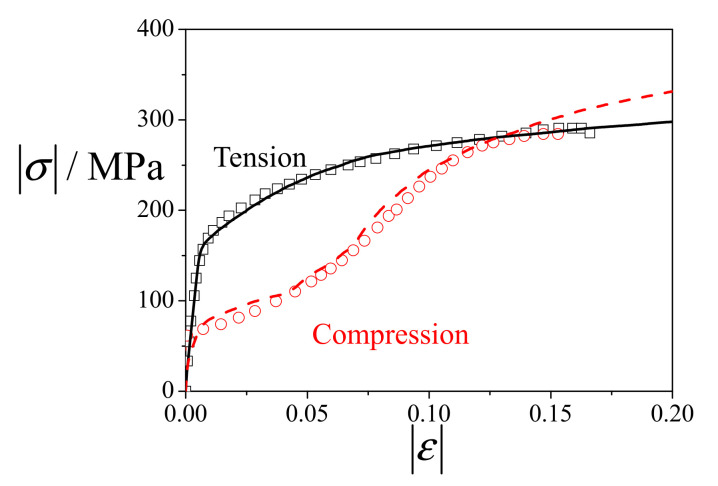
Experimental (symbols) and simulated (lines) stress–strain curves of uniaxial tension and compression along the RD.

**Figure 3:**
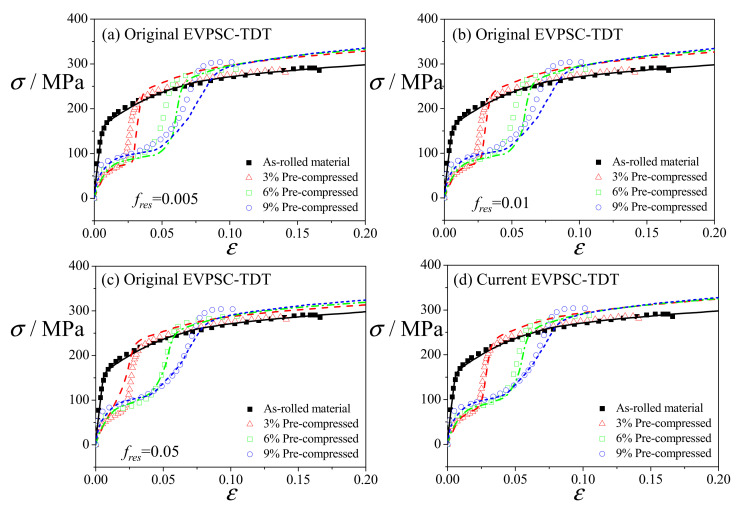
Experimental (symbols) and simulated (lines) stress–strain curves under uniaxial tension along the RD. The numerical results were obtained by using the original EVPSC-TDT model with constant t value for $f_{res}$ of 0.005 (**a**), 0.01 (**b**) and 0.05 (**c**) and the current EVPSC-TDT model (**d**). The experimental data are from Park et al. [1].

The values of the material parameters were determined by curve-fitting numerical simulations of monotonic tension and compression along the RD to the corresponding experimental data. Figure 2 presents the fitted and experimental stress–strain curves along the RD. The tensile stress–strain curves exhibit a typical slip-dominated convex shape, while the compressive stress–strain curves show a twin-induced sigmoid shape. It is seen that the EVPSC-TDT model can reproduce the experimental data well. The material parameters listed in Table 1 were used in all the simulations. Regarding the parameters for detwinning (Equation (2)), we referred to the work by Yu et al. [24,25]. However, their reported parameters were used for a single Mg crystal under cyclic loading. Here, the parameters $a_{twin}$ and $b_{twin}$ for detwinning were mainly determined by curve-fitting numerical results of the 3% pre-compressed material under reverse tension (shown in Figure 3b). As reported by Lou et al. [17], the residual twin volume fractions of AZ31 alloys with 1%, 3% and 7% pre-compression under reverse tension to a tensile strain of 6% are ~3%, ~2% and ~9%, respectively. Therefore, the value of $a_{twin}$, i.e., $a_{twin}$ = 0.05, seems reasonable based on the experiments. In addition, it is assumed that the proposed modeling strategy of detwinning behavior is more closely related to the amount of pre-induced twins (see, Equation (2)) rather than the applied loading direction during pre-straining; however, experiments with pre-compression and reverse tension applied along the directions other than the RD are actually desired for further evaluation of the predictability of the calibrated model.

The predicted initial textures of 3% pre-compressed, 6% pre-compressed and 9% pre-compressed materials are shown in Figure 1b–d. Note that these initial textures are the textures of the as-received plate deformed under uniaxial compression along the RD at a strain of 3%, 6% and 9%, respectively. These results are in good agreement with the textures measured by Park et al. [1]. As shown in Figure 1b–d, a large portion of basal poles is observed to align around the RD after the pre-compression, which is caused by the twinning-induced lattice reorientation. The initial grain orientations of the pre-compressed materials can be divided into two groups: one with their *c*-axis approximately parallel to the ND direction (residual matrix) and the other with their *c*-axis approximately parallel to the RD direction (RD twin). The basal texture in the residual matrix becomes weak with the increase of the amount of pre-compression.

The original EVPSC-TDT model employs a constant value for $f_{res}$ to constrain detwinning in a grain [35]. Figure 3a–c present the experimental and simulated stress–strain curves under uniaxial tension along the RD for the as-rolled and pre-compressed materials by using the original EVPSC-TDT with various constant values for $f_{res}$ of 0.005, 0.01 and 0.05. It is clear that by using a constant value for $f_{res}$, the measured stress–strain curves cannot be well re-produced numerically for all the three pre-compressed materials. In contrast, with the proposed Equation (2) for the residual twin, the modified EVPSC-TDT model provides a much better prediction, as shown in Figure 3d. The as-rolled material with the *c*-axis aligned parallel to the ND is subjected to the loading condition of tension perpendicular to the *c*-axis, and this is unfavorable for the occurrence of the {10-12} twins. The flow curve, therefore, exhibits a concave down shape, which is a typical feature of slip-dominated deformation. For the pre-compressed materials, the $\left\{10\bar{1}2\right\}$ extension twins produced in pre-compression will facilitate the detwinning during reverse tension along the RD, resulting in a typical sigmoid shape of flow curves.

For the 3% pre-compressed material, the calculated twin volume fraction (~29%) is larger than the experimental value of ~19%. Detwinning dominates during the early stage of tensile deformation, and the yield stress is lower than that in as-rolled materials due to low activation stress for detwinning. Since the twin volume fraction formed during pre-compression is small, detwinning is exhausted at the strain of ~3%, which corresponds to the inflection point in the flow curve. After detwinning is exhausted, slips become the dominated deformation mechanisms, leading to a rapid increase of the flow stress with a concave down shape [41]. The simulated flow curve is slightly overestimated at large strains. For the 6% pre-compressed material, the predicted twin volume fraction is ~56%, while the experimental value is 52%. The early deformation is still dominated by detwinning, causing a sigmoid shape of the flow curve. The predicted inflection point, reflecting the exhaustion of detwinning, occurs at a strain of ~5%, slightly larger than the experimental one. After detwinning is exhausted, slips become the dominated deformation modes, and the simulated flow curve is slightly underestimated. For the 9% pre-compressed material, the measured twin volume fraction is ~76%, larger than the predicted one (~70%). The deformation mechanisms are the same as those in 3% and 6% pre-compressed materials. The inflection point occurs at a strain of ~8%.

It should be pointed out that the tensile yield stress of pre-compressed materials is much lower than that of the as-rolled material due to the change in the yield mechanism from the prismatic slip to detwinning, in which the CRSS is much lower. A gradual increase of the tensile yield stress with the pre-compression was reported by Park et al. [1]. The authors mentioned that this was mostly due to the dislocation-interaction-related hardening caused by the pre-compression. Lou et al. [16,17,18] also investigated the effect of twin lamellar structure produced by pre-compression on the yield strength and ductility of magnesium alloys under reverse tension and found that the increase of tensile yield stress with the pre-compression was mostly caused by the detwinning-induced texture hardening rather than the Hall–Petch mechanisms.

**Figure 4:**
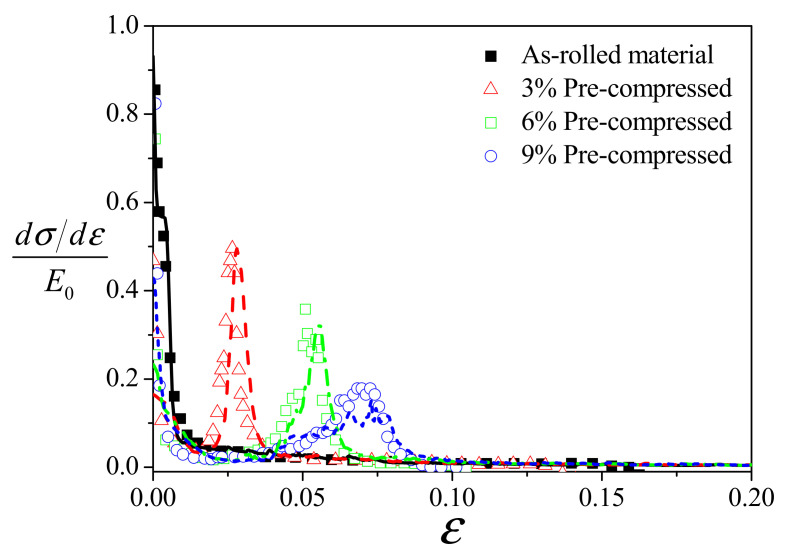
The measured (symbols) and predicted (lines) normalized strain hardening rate versus strain under uniaxial tension along the RD. The experimental data are from Park et al. [1].

The predicted and experimental curves of the normalized strain hardening rate θ($=\frac{d\sigma /d\varepsilon }{E_{0}}$) as a function of true strain are shown in Figure 4 ($E_{0}$ = 45 GPa). The predicted results match the corresponding experimental data very well. It should be noted here that the strain hardening rate is calculated from the true stress–strain curves. For the as-rolled material, the normalized strain hardening rate decreases rapidly from ~1.0 to ~0.06 with the strain up to ~0.01; after that, it gradually evolves at a small rate, showing a characteristic of slip-dominated deformation. For the pre-compressed materials, an inflection point between stages B and C (see, [13]) could be clearly found, indicating a transition of deformation mechanisms from detwinning dominance to slip dominance. The peak value of the normalized strain hardening rate corresponding to the inflection point decreases when increasing the amount of pre-compression from 3% to 9%, and the corresponding strain is proportional to the amount of pre-compression (also see [19]).

**Figure 5:**
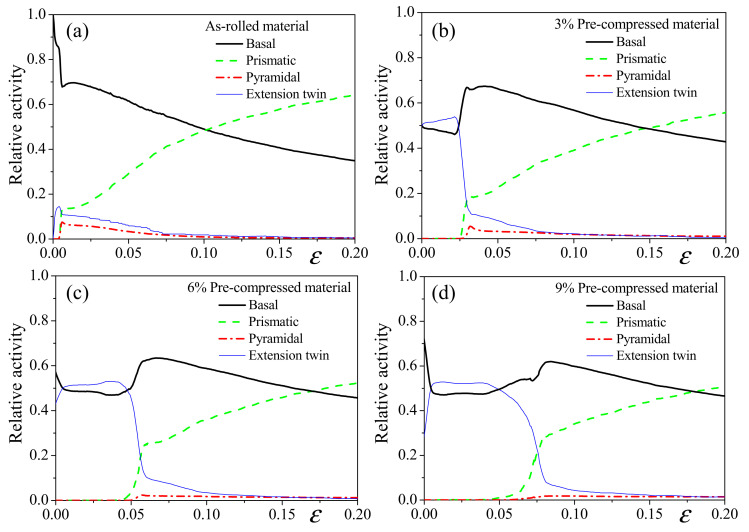
Predicted relative slip/twinning activities under uniaxial tension along the RD of: (**a**) as-rolled, (**b**) 3% pre-compressed, (**c**) 6% pre-compressed and (**d**) 9% pre-compressed materials.

Figure 5 provides the predicted relative activities of slips and tensile twinning of the as-rolled and pre-compressed materials under tension along the RD. For the as-rolled material, basal slip dominates up to a strain of ~0.1, while prismatic slip becomes dominant with further straining. Small twinning activity could also be found in the early stage of deformation because of initial imperfect texture. For the pre-compressed materials, irrespective of the amount of pre-compression, basal and detwinning accommodate the total plastic deformation until prismatic slip is activated with the exhaustion of detwinning. Moreover, it is obvious that the exhaustion process of detwinning lasts longer when increasing the amount of pre-compression. In addition, pyramidal slip is almost non-activated in the pre-compression materials, while it is activated during the early deformation of the as-rolled material.

**Figure 6:**
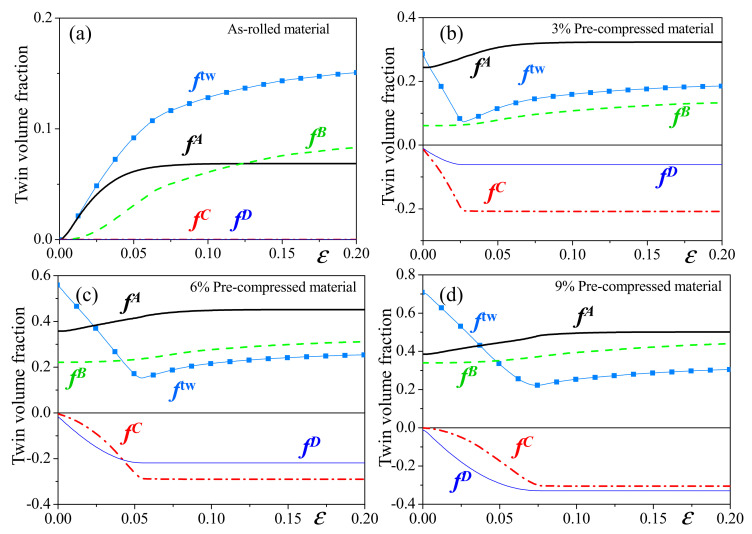
The predicted evolution of twin volume fractions under uniaxial tension along the RD of: (**a**) as-rolled, (**b**) 3% pre-compressed, (**c**) 6% pre-compressed and (**d**) 9% pre-compressed materials.

Figure 6 presents the net evolution of twin volume fraction ($f^{tw}$) and the accumulated twin volume fractions associated with Operations A, B, C and D as a function of the strain of different materials under tension along the RD. For the as-rolled material, as previously mentioned (see in Figure 5a), twinning is slightly activated due to the imperfect texture. The twin volume fraction at the end of deformation is ~15%. For the pre-compressed materials, regardless of the amount of pre-compression, the twin volume fraction ($f^{tw}$) decreases in the early stage of deformation due to the activity of detwinning ($f^{C}$,$f^{D}$). The accumulated twin volume fractions related to Operations C and D (detwinning) increase during the early deformation stage and saturate after ~3%, ~5% and ~8% strain for the 3%, 6% and 9% pre-compressed materials, respectively. The saturated volume fraction of detwinning increases when increasing the amount of pre-compression. Note that the twin volume fraction exhibits a small increase even after the exhaustion of detwinning, which should be caused by the imperfect texture and slip-induced reorientation.

**Figure 7:**
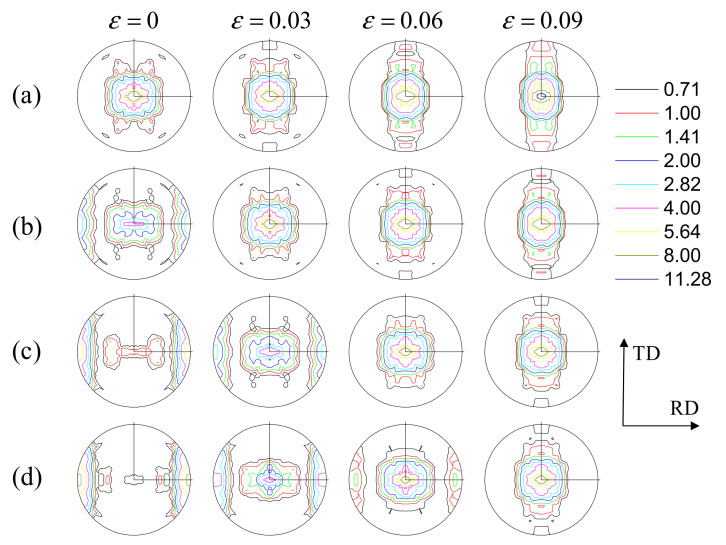
{0002} pole figures of: (**a**) as-rolled, (**b**) 3% pre-compressed, (**c**) 6% pre-compressed and (**d**) 9% pre-compressed materials under tension along the RD to different stain levels.

Figure 7 shows the texture evolutions of the as-rolled, 3% pre-compressed, 6% pre-compressed and 9% pre-compressed materials under tension along the RD to a strain of 0.03, 0.06 and 0.09. As mentioned by Park et al. [1], the RD twin texture, generated by the pre-compression, was almost completely restored to the ND initial texture through detwinning, which is reflected well in our simulation results (see Figure 7b–d). From Figure 7b, it is seen that the RD twin texture, generated by the pre-compression in 3% pre-compressed materials, is almost completely restored to the ND initial texture through detwinning at the strain of 0.03, which is consistent with the experimental observation (see, [1]). For the 6% and 9% pre-compressed materials (Figure 7c,d), the RD twin texture, is not completely restored at the strain of 0.03; however, it can be seen that the RD twin textures in the 6% and 9% pre-compressed materials are completely restored to the ND initial texture at the strain of 0.06 and 0.09, respectively. It is demonstrated again that the detwinning process is closely related to the amount of pre-compression.

## 4. Conclusions

In this study, the EVPSC-TDT model was employed to study the effect of pre-strain on detwinning in a rolled Mg alloy AZ31 under reverse tension along the RD. Compared with the as-rolled material, the pre-compressed materials exhibit different mechanical behavior due to the activation of detwinning. A new criterion was proposed to control the exhaustion of detwinning of the pre-compressed materials under reverse tension. Our numerical results are in good agreement with the corresponding experimental data. It was demonstrated that the proposed criteria can capture the key features associated with detwinning in the pre-compressed materials under reserve tension. The tensile yield stresses of pre-compressed materials are much lower than that of as-rolled material due to the low activation stress of detwinning. The inflection point in flow curves showing the exhaustion of detwinning is delayed with increasing the amount of pre-compression. The peak value of normalized strain hardening rate decreases with an increasing amount of pre-compression. The RD twin textures in the 3%, 6% and 9% pre-compressed materials are completely restored to the initial ND texture at the strains of 0.03, 0.06 and 0.09, respectively. In addition, with the proposed criterion, the EVPSC-TDT model should also be applicable for simulation of the detwinning behavior of Mg alloys upon reversed shear tests, e.g., [34,42], which deserves further investigation.

## Figures and Tables

**Table 1:** List of values of the material parameters determined by fitting the monotonic uniaxial tension and compression stress–strain curves of the as-received AZ31 plate. $h^{st}$ represents the latent hardening coefficients of slip systems associated with the twining systems.

Mode	$\tau_{0}(\mathrm{MPa})$	$\tau_{1}(\mathrm{MPa})$	$h_{0}(\mathrm{MPa})$	$h_{1}(\mathrm{MPa})$	$h^{st}$	$B_{1}$	$B_{2}$
Basal	7	1	10	10	1		
Prismatic	88	25	700	5	2		
Pyramidal	90	90	500	0	1		
Extension twin	15	10	50	10	1	0.7	0.85
(detwinning)		$a_{twin}=0.05$	$b_{twin}=0.1$				

## Data Availability

Data sharing is not applicable.
